# Random Insertion Reporter Gimmicks Powered by Cut-and-Paste DNA Transposons

**DOI:** 10.3390/biomedicines13071682

**Published:** 2025-07-09

**Authors:** Yamato Kasahara, Kentaro Semba, Shinya Watanabe, Kosuke Ishikawa

**Affiliations:** 1 Department of Life Science and Medical Bioscience, Waseda University, 2-2 Wakamatsu-cho, Shinjuku-ku, Tokyo 162-8480, Japan; y.k4309@akane.waseda.jp (Y.K.); ksemba@waseda.jp (K.S.); 2 Translational Research Center, Fukushima Medical University, 1 Hikarigaoka, Fukushima 960-1295, Japan; 3 Japan Biological Informatics Consortium (JBiC), 2-45 Aomi, Koto-ku, Tokyo 135-8073, Japan

**Keywords:** DNA transposon, reporter, random screening, transposase

## Abstract

Transposons are mobile genetic elements capable of moving within the genome. Leveraging this property—particularly the cut-and-paste mechanism of DNA transposons—has enabled the development of technologies for inserting exogenous DNA fragments into host genomes. While targeted integration is a key goal for therapeutic applications, this review highlights the value of their intrinsic randomness. By combining the ability to freely design the DNA cargo with the stochastic nature of transposon integration, it becomes possible to generate highly sensitive reporter cells. These can be used to efficiently identify functional markers, uncover novel signaling pathways, and establish innovative platforms for drug screening. As more subfamilies of transposons become available for research use, their complementary biases may enhance the coverage and diversity of genome-wide screening approaches. Although inherently unpredictable, this strategy embraces randomness as a strength, and we propose that it holds great promise for driving new advances in biology, cellular engineering, and medical research.

## 1. Introduction

Transposons, or mobile genetic elements, were first described by Barbara McClintock in her seminal work “The origin and behavior of mutable loci in maize”. These elements are capable of moving within the genome, a finding that laid the foundation for the field of mobile DNA and profoundly influenced our understanding of genome dynamics [1,2]. Transposons constitute a substantial portion of the genome, and some play critical roles not only in regulating gene expression but also in altering the DNA sequences of genes themselves. In certain cases, transposons have been “domesticated” by the host genome, serving as a driving force for genetic innovation and phenotypic diversity [3].

Among the various types of transposons that have been studied, autonomous cut-and-paste DNA transposons represent one of the most widely utilized families in experimental research [4]. This family encodes an enzyme called transposase, which recognizes terminal sequences within the transposon and excises the entire element as a discrete DNA fragment. The transposase then mediates the integration of this fragment into a new genomic location. Remarkably, this single multifunctional enzyme enables the efficient and straightforward delivery of DNA fragments—without strict limitations on cargo size—into the genome [5,6,7,8,9,10]. Compared to viral vector systems, DNA transposon-based approaches offer several advantages, including applicability to non-dividing cells and the development of marker-free vector systems [11,12,13]. Additional improvements—such as strategies to prevent the inclusion of toxic elements [14,15] and the inherently low immunogenicity of transposons [16], which has been further minimized—have addressed many safety concerns. Moreover, the use of mRNA to transiently express the transposase adds another layer of control, further reducing potential risks associated with clinical applications [17,18,19,20,21,22,23].

As a result, DNA transposons are being actively developed as highly promising vectors for clinical applications. In particular, their use in chimeric antigen receptor T-cell (CAR-T) therapies has attracted significant attention [23,24,25,26,27,28].

In recent years, targeting technologies that add specificity-conferring functional domains, such as RNA-guided DNA endonucleases (e.g., Cas9, Cas12, and Fanzor), to cut-and-paste transposases have garnered significant attention [29,30,31,32,33]. These innovations are seen as promising approaches for achieving ideal gene delivery with minimal off-target effects, embodying the potential for precise genetic manipulation.

Nonetheless, it is important to revisit the inherent property of cut-and-paste transposons that necessitates the use of such specificity-conferring technologies. This property is the ability of transposons to insert arbitrary DNA relatively randomly into the genome. The utilization of this randomness has proven valuable in techniques such as insertional mutagenesis and forward genetics, playing a crucial role in the discovery of genes implicated in various diseases (described in Section 3.3).

What is less widely recognized, however, is that the true potential of these systems can be further unlocked by designing DNA sequences or refining screening protocols, which significantly enhances their utility. Through the careful design of both the DNA sequences and screening protocols, we have demonstrated, in several proof-of-concept studies, not only an improvement in the sensitivity of random screening using reporter genes, but also the potential to further enhance their effectiveness and create new biotechnological innovations.

In this review, we briefly and comprehensively discuss examples of the research applications of cut-and-paste DNA transposons, while highlighting the potential of utilizing randomness as a powerful approach. We aim to convey how this strategy can play a critical role in identifying targets and markers that are difficult to discover using techniques such as microarrays or next-generation sequencing. Furthermore, we explore how this approach may lead to unexpected breakthroughs in long-standing research problems and offer new insights, potentially driving innovative advancements in drug development. While the use of randomness is often perceived as a gamble or based on luck, in practice, the frequency of hitting a relevant gene is not low, and we wish to emphasize the value and significance of this approach. This strategy, though bold in its embrace of unpredictability, has the potential to pave the way for new advancements in future biotechnology, and we are confident it will open new avenues for progress.

## 2. Cut-and-Paste DNA Transposons: Structural Overview and Applications in Research

### 2.1. TIR

Terminal inverted repeats (TIRs), located at both ends of the transposon and defining its junctions with the host genome, should ideally be selected based on their minimal influence on the host—preferably lacking promoter or enhancer activity. At present, such ideal selection is challenged by the limited availability of thoroughly studied transposons, yet future research may substantially broaden these possibilities.

### 2.2. Enzymatic Activity of Transposases Used in Research

**Figure 1:**
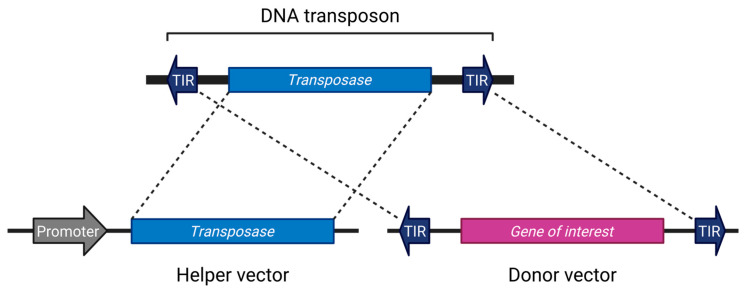
The general DNA transposon system. Created in BioRender (https://BioRender.com/vduuzsw (accessed on 7 July 2025)).

Although there are exceptions [34], most transposon systems used in research are divided into two components: a helper vector expressing the transposase, and a donor vector containing the gene of interest flanked by terminal inverted repeats (TIRs) [4,35,36,37,38,39]. This configuration is reminiscent of the structure of early transposons such as Ds and Ac (Figure 1). The current mainstream systems employ a single transposase protein capable of excising, mobilizing, and inserting the transposon without reliance on host factors [40]. However, there is no fundamental reason to restrict applications to this format; systems involving two cooperating open reading frames (ORFs) have also been utilized [33,41,42]. Separating the transposase from the donor vector can help prevent unintended remobilization [43].

Once the donor vector is appropriately engineered, the transposon system can autonomously carry out precise cut-and-paste integration into the host genome, thereby minimizing the need for further intervention.

### 2.3. Sequence-Specific Targeting Bias of Transposases

Each transposase recognizes a unique target site sequence (TSS), with examples such as *PiggyBac* (PB): TTAA [44], *Sleeping Beauty* (SB): TA [45], and *Tol2*: AT-rich [46]. As a result, each transposase exhibits distinct preferences for target sites. Additionally, the specificity of integration is influenced not only by the target sequence itself but also by factors such as the host cell type, the epigenetic state of the genome, and higher-order chromatin structures, including chromatin loops [47,48,49,50,51,52,53,54,55]. These factors contribute to a sequence-specific targeting bias, reflecting the transposase’s preference for certain genomic contexts beyond the core recognition motif. Therefore, as demonstrated in the example by Elling et al. (2017) [56], screening with multiple transposases or using different cell lines with distinct characteristics can enable more comprehensive exploration of genomic regions. Each transposase complements the others in terms of their target preferences.

Comparisons have also been made with viral systems [57,58,59,60,61]. For instance, lentivirus tends to preferentially integrate into transcriptionally active regions, a process associated with its binding to the ubiquitous nuclear protein LEDGF/p75 [62]. Therefore, when transposons are used for screening, they are generally considered to result in less overlap compared to viral vectors [63,64]. After integration, transposons can influence gene expression by exerting cis-acting effects on nearby regulatory elements such as promoters, enhancers, and suppressors, or by causing gene fusions. However, their transcriptional activity is much lower than that of viral vector LTRs, and thus they exert minimal transcriptional impact on surrounding genes [65]. By utilizing stable expression, transposons provide a method for introducing genes with minimal unwanted sequences, which is particularly advantageous for the production of recombinant therapeutic proteins (RTPs) in antibody-based therapies. Mammalian cells such as CHO and 293T cells are commonly used for this purpose [66,67,68,69].

### 2.4. Improvement of Transposase

The higher the activity of transposase, the greater the efficiency of gene delivery. Ongoing efforts include the exploration of alternatives for improved efficiency, studies on high-activity mutations, and the development of refined control methods [70,71,72,73,74,75,76,77,78,79]. More recent examples include Passer [80] and Mariner2_AG (MAG) [40]. The reanalysis of the PS family’s distribution and evolution, combined with cell-based assays, identified Passer (PS) transposons from *Gasterosteus aculeatus* and *Danio rerio* as novel elements with exceptionally high activity in human cells, surpassing Sleeping Beauty (SB) [80]. Genome-wide in silico screening identified candidate autonomous DNA transposons with intact transposase ORFs and terminal inverted repeats, predicted to be active based on target site duplications and low sequence divergence. Among them, MAG demonstrated superior performance to lentiviral vectors in CAR-T cell therapy [40].

The search for yet undiscovered transposases is expected to continue in the future.

## 3. Randomness and Its Value

Transposons have played a significant role in the evolution of life by introducing genomic alterations subject to natural selection over vast evolutionary timescales. Harnessing this inherently sophisticated mechanism of genome remodeling provides a powerful strategy for modern biological research. Their capacity to induce widespread and stochastic changes in the genome aligns with a broader principle in scientific discovery: that randomness, when coupled with systematic observation, can yield profound insights.

In general, large-scale screenings have led to numerous groundbreaking discoveries that were seemingly brought about ‘by chance’. Countless similar examples can be found throughout the history of science, and they demonstrate that such outcomes are not mere coincidences, but rather reflect effective research strategies. Accordingly, when using transposons in research, it is important to actively incorporate their inherent ability to scan broad genomic regions in a relatively unbiased and random manner [81,82].

### 3.1. Historical Development and Impact of Transposon Randomness

Transposons have long been recognized not only for their ability to integrate into genomes, but also for their intrinsically random insertion patterns. Since the early days of genetic research, this stochastic behavior has been purposefully exploited to uncover gene functions, map regulatory elements, and conduct large-scale mutagenesis.

In eukaryotic systems, the field progressed rapidly with pioneering work in *Drosophila melanogaster*, where P elements were engineered for gene trap strategies [83,84,85,86]. These modified transposons enabled researchers to simultaneously induce mutations and tag disrupted genes, greatly facilitating the identification of insertion sites. As a result, transposons emerged as “tagged mutators”—genetic tools capable of both introducing mutations and leaving a traceable molecular footprint.

This innovation transformed functional genomics by making forward genetic screening more efficient and informative. From early work in *Drosophila* and maize to modern genome-wide insertional screens in mammalian cells, the inherent randomness of transposons has consistently served as a powerful and unbiased approach to gene discovery.

### 3.2. Transition to Mammalian Systems

The successful use of DNA transposons in *Drosophila* for studying development and signaling pathways provided a strong impetus for applying this technology in mammalian systems. However, transposition of P element requires factors that are absent or insufficient in mammalian cells, posing a significant barrier to its direct application in such systems. Similarly, plant-derived elements like the Ac transposon, originally identified in maize and also used in rice and morning glory (*Ipomoea*), proved effective within plant systems [87] but failed to function efficiently in mammalian contexts. Therefore, realizing transposon-based mutagenesis in mammals—especially for somatic and germline applications in model organisms like the mouse—required critical breakthroughs to overcome these cross-species functional limitations.

The breakthrough came with the introduction of transposon systems such as *piggyBac* [88,89,90,91], *Sleeping Beauty* [73,92], and *Tol2* [17,93], which were found to be active across a broad range of species, including humans and mice [81,94]. Their demonstrated ability to transpose efficiently in diverse biological contexts marked a turning point, leading to their rapid adoption as versatile tools for gene delivery. These systems have since become central to transposon-based technologies, particularly due to their broad host compatibility and robust integration activity. Given the wide and growing body of work on these systems, readers are referred to comprehensive reviews for further details [4,74].

### 3.3. Transposon Applications in Vertebrate Species: Advances in Mammalian Mutagenesis

The utilization of these transposon systems has enabled long-awaited success in performing forward genetics in mice—where phenotypic observations are followed by the identification of the underlying gene(s). It has been demonstrated that germline transgenesis can be achieved, followed by subsequent transposition events, thus establishing a powerful platform for genetic analysis in mammalian models [81,95,96,97,98,99]. Although there was a period when transposition efficiency was insufficient for practical applications, this limitation has since been overcome through advances such as the hyperactivation of the *Sleeping Beauty* system, as well as the optimization and inducible control of transposition in the *piggyBac* system [100].

With the achievement of practical levels of transposon-based insertional mutagenesis, it became possible to perform screening for oncogenes and tumor suppressor genes in mouse models [101,102]. Screening for essential genes, as well as cancer-related genes [103] such as whole-body solid cancers [104], was successfully conducted. Additionally, tissue-specific cancer models, such as mouse hepatocellular carcinoma (HCC) [105], squamous cell carcinoma [106], hematopoietic tumors [107], and colon cancer (CRC) [108], were investigated. Furthermore, the *Sleeping Beauty* system enabled the identification of genes involved in the progression of CRC [109]. By utilizing Blm-deficient ES cells, which enhance DNA damage and recombination error efficiency, screening for mismatch-repair (MMR) genes was shown to outperform retrovirus-based screening [110]. Additionally, there is research that elucidated the mechanism by which transposon-induced mutations convert neural stem cells into glioma-initiating cells [111]. Also, transposon insertional mutagenesis has been applied to hematological malignancies, such as leukemia, to identify key genetic drivers of disease progression [112,113]. Furthermore, this method has also demonstrated utility beyond cancer research, including in the investigation of metabolic disorders such as diabetes [114].

Through research utilizing random insertion, many breakthroughs have been achieved in understanding gene function and signaling pathways. Simultaneously, transposons with high utility value have been identified, and their potential for medical applications has become evident. As a result, the status of transposon-based cut-and-paste technology has become firmly established.

## 4. Gene Screening Utilizing Randomness: Proof-of-Concept Studies Based on Our Transposon-Mediated Approaches

By modifying the DNA of the donor vector, it is possible to control the fusion with endogenous genes. We have developed several screening systems that take advantage of the inherent randomness of transposons

### 4.1. Fusion of Genes with Reporter Proteins: Applications in Protein–Protein Interaction Analysis

Through transposon-mediated genome delivery, it is possible to fuse reporter proteins, such as fluorescent proteins, with endogenous genes, enabling the spatiotemporal monitoring of cells, protein–protein interactions, and localization (all at the protein level).

**Figure 2:**
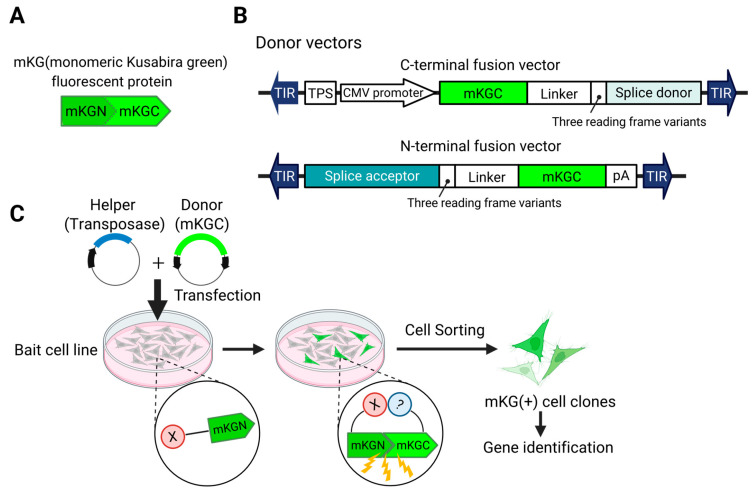
Transposon system for identifying genes encoding interacting proteins. (**A**). To induce BiFC (bimolecular fluorescence complementation), a fluorescent protein (in this case, mKG) is split into N- and C-terminal fragments. The N-terminal fragment is fused to the protein of interest (in this case, X) to generate the Bait strain. (**B**). Donor vectors are constructed to fuse the C-terminal fragment (mKGC) to endogenous genes. (**C**). Workflow: The donor vector and a helper vector encoding a transposase are co-transfected into the bait cell line. When an mKGC-fused protein interacts with the bait, the proximity of mKGC to mKGN leads to the reconstitution of mKG, resulting in fluorescence. This allows the isolation of cells in which the vector has been inserted into the gene encoding the interacting protein.

We have established a screening system for binding proteins by modifying transposon DNA to enable fluorescence recovery through the reassembly of fluorescent proteins via the BiFC phenomenon [115] (Figure 2). In this method, a fluorescent protein (e.g., GFP) is split into two non-fluorescent fragments, each fused to one of the two proteins whose interaction is to be investigated. When these proteins interact within the cell, the fragments of the fluorescent protein are reassembled, and a fluorescence signal is generated. By observing the presence or absence of fluorescence, protein–protein interactions can be confirmed, allowing for screening and separation. Unlike traditional methods that require the creation of a library, each screening can be performed with a unique library for each batch. Using this approach, we were able to discover and rediscover binding factors of NF-κB [115].

### 4.2. Fusion of Promoters/Enhancers with Reporter Genes

This technology uses a vector, which integrates randomly into a genome and is designed to express a reporter gene driven by the near cis-acting promoter/enhancer elements [116,117]. Although the target gene is left to the randomness of transposon insertion, this reporter cell generation method is simpler than attempting to design the integration site deliberately. Previously, retrovirus-based gene (or promoter) trap methods were widely used. However, compared to retroviral systems, transposons allow for a more flexible DNA sequence design and represent one of the most powerful approaches for generating reporter cells. While many promoter trap methods using fluorescent protein reporters have been developed in the past [118,119], they often suffered from low sensitivity, making it difficult to isolate reporter-positive cells from a large population of negatives.

**Figure 3:**
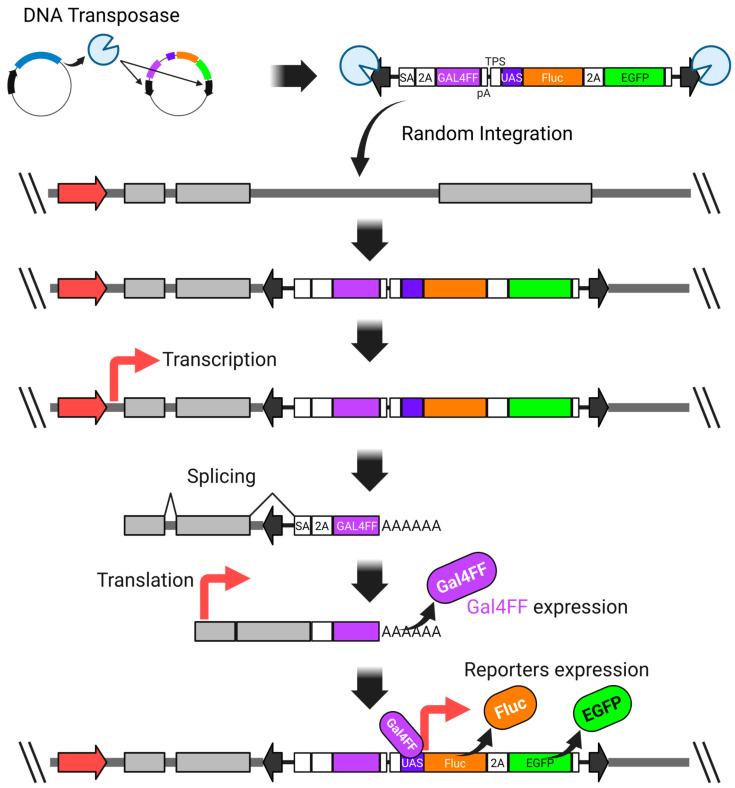
A highly sensitive promoter-trap vector system utilizing both the Gal4-UAS expression system and the *piggyBac* transposon system. SA—synthetic splicing acceptor; P2A—2A peptide derived from porcine teschovirus-1; GAL4FF—an extremely trimmed minimal DNA-binding site of the yeast GAL4 transcription factor with a few repeats of the minimal transcription activation module from VP16; polyadenylation signal; TPS—transcription pause site; UAS—upstream activation sequence; Fluc—firefly luciferase; EGFP—enhanced green fluorescent protein. The thick red arrow represents an endogenous promoter.

We overcame this issue by incorporating the GAL4FF-UAS system into the donor vector, which significantly enhanced the detection sensitivity. GAL4FF is a modified form of the yeast Gal4 transcription factor, consisting of a highly truncated DNA-binding domain and tandem repeats of a minimal transcription activation module derived from VP16 [116,117,120,121,122]. This engineered transcription factor enables robust and specific activation of UAS-linked reporter genes (Figure 3).

Such an approach would not have been possible without the unique advantage of transposons: their ability to deliver DNA sequences without significant constraints on size or content.

### 4.3. High-Sensitivity Trap Vector-Based Random Screening

**Figure 4:**
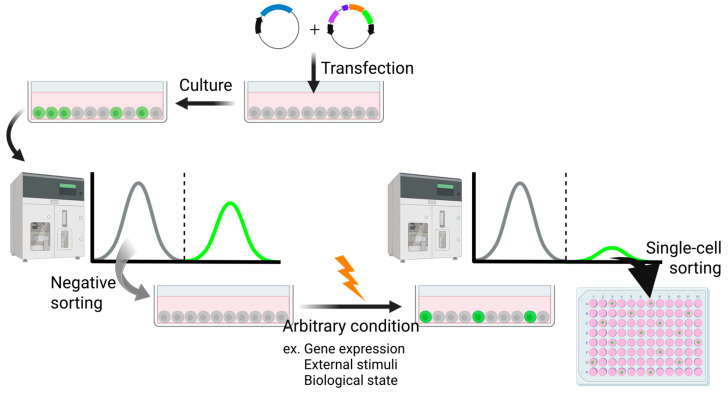
An experimental procedure using the transposon system to yield reporter cells responsive to arbitrary conditions. Created in BioRender (https://BioRender.com/mikk94m (accessed on 7 July 2025)).

The implementation of a high-sensitivity trap vector has substantially broadened the scope of reporter cell generation, rendering it applicable under diverse experimental conditions (Figure 4). This system is capable of detecting subtle transcriptional changes that often remain undetected in conventional approaches such as microarray-based profiling. Notably, it facilitates the identification of previously unrecognized marker genes, thereby enabling an unbiased and sensitive screening strategy.

#### 4.3.1. Isolation of c-Myc-Responsive Cells

The response conditions for reporter cells are not limited to pharmacological stimuli; rather, they can theoretically include any biological or environmental state, such as cellular stress or differentiation status. This flexibility has enabled the establishment of highly versatile reporter cell systems. Among these diverse conditions, gene expression itself can also serve as a trigger. We established a cell line with inducible c-Myc expression, from which c-Myc-responsive cells were successfully isolated using our engineered transposon-based vector system [116]. Furthermore, these c-Myc-responsive reporter cells were employed for screening small-molecule inhibitors, leading to the identification of novel compounds capable of suppressing c-Myc activity [123,124].

#### 4.3.2. Isolation of Cells Responsive to ER Stresses

We further applied our strategy to isolate reporter cells responsive to well-characterized physiological conditions such as endoplasmic reticulum (ER) stress [116]. Reporter-positive cells were isolated following treatment with ER stress-inducing agents, such as thapsigargin and tunicamycin. Upon analyzing the genes fused to the reporter, we successfully identified BiP, a canonical ER stress marker, thereby demonstrating that classical markers can be readily recovered even through an entirely random screening approach.

In addition to known markers, we also discovered previously unreported genes, such as *OSBPL9*, which had not been associated with ER stress responses. Notably, *OSBPL9* had ranked low in conventional microarray-based differential expression analyses, highlighting the limitations of ranking-based methods in capturing subtle but biologically relevant changes. Our random insertion-based screening method proved effective in identifying such elusive markers, which are often missed by traditional approaches.

Moreover, this strategy led to the identification of a novel class of genes, including *TISPL* (transcript induced by stressors from *LINC-PINT* locus) [125], which appears to be transcribed from intronic regions of host genes. These loci are not annotated in current genome databases and would be extremely difficult to predict using conventional gene-finding algorithms. Our findings, therefore, validate the utility of leveraging randomness in genetic screening, not only to identify known responsive genes, but also to uncover novel transcripts arising from unanticipated genomic loci and regulatory contexts.

#### 4.3.3. Cross-Pathway Compound Profiling Using Randomly Generated Reporter Cell Lines for Vitamins and Forskolin

Using the same transposon-based reporter vector system, we next performed random screening to isolate cells responsive to bexarotene (a vitamin A analog) and calcitriol (an active form of vitamin D) [126]. Remarkably, despite the stochastic nature of the screening, *CYP24A1*, a well-established target gene of vitamin D signaling, was successfully isolated. In addition, we identified two novel vitamin-responsive genes: *BDKRB2*, which responded specifically to bexarotene, and *TSKU*, which responded to both bexarotene and calcitriol, indicating that *TSKU* is a multi-vitamin-responsive gene.

Further analysis of multiple reporter cell clones obtained through this screening revealed that each exhibited a distinct and reproducible transcriptional response profile upon treatment with various vitamins. Quantitative assessment of reporter activity across these clones showed that each vitamin elicited a unique pattern of activation, thus enabling the discrimination of compound-specific responses.

These findings demonstrate that random insertional reporter systems can not only recover canonical markers but also uncover previously unrecognized, functionally relevant genes. Moreover, by leveraging a panel of such reporter clones, it becomes possible to perform functional compound profiling, providing a powerful platform for characterizing the biological specificity of small molecules such as vitamins.

As the most striking example, we discovered that, by utilizing multiple forskolin-responsive reporter cell lines, it was possible to profile tyrosine kinase inhibitors, which would otherwise appear to be mechanistically unrelated to cAMP signaling pathways [127]. This unexpected cross-pathway responsiveness suggests that compound profiling using diverse reporter cell lines can reveal hidden pharmacological relationships and off-target effects, thereby enhancing the utility of this system for drug discovery and mechanistic exploration.

#### 4.3.4. Reporter Cells Revealing Hidden Cell Cycle Dynamics

During the generation of reporter cells using our trap vector system, we routinely eliminated clones that exhibited constitutive reporter expression prior to the application of any external stimulus. However, even after multiple rounds of negative sorting to eliminate cells with pre-induced reporter activity, we consistently failed to remove a subset of cells exhibiting persistent reporter expression. Further analysis revealed that some of these cells were expressing the reporter in a cell cycle-dependent manner (our unpublished data).

This serendipitous finding implies that our screening system, initially designed for random trapping, can also capture dynamic gene expression linked to intrinsic cellular processes such as the cell cycle. More importantly, it suggests that a wide array of novel cell cycle markers may be discovered through this approach—markers that may have remained undetectable through conventional methods.

This insight opens a new avenue of research inspired by the intrinsic temporal regulation of gene expression, and highlights the versatility of our reporter system in uncovering fundamental biological rhythms.

## 5. Conclusions

Our generation inherits the fruits of decades of dedicated research by scientists who sought to understand the nature of transposons. Among them, the use of class II (DNA-type) “cut-and-paste” transposons stands out as particularly powerful. Their defining property—the ability of transposases to integrate freely designed DNA sequences into the genome in an almost random manner, without limitations on insert size—offers immense utility.

Like a sculptor wielding a chisel to reveal the potential hidden within stone, this foundational technology empowers creative ideas and innovative concepts to flourish. In our study, we demonstrated several proof-of-concept examples using randomly generated reporter cells, primarily based on stable cell lines. Through this approach, we successfully rediscovered known markers and binding proteins with high sensitivity, while also uncovering entirely novel genes. These results underscore the tremendous versatility and power of the transposon system, particularly when coupled with a strategic embrace of randomness.

**Figure 5:**
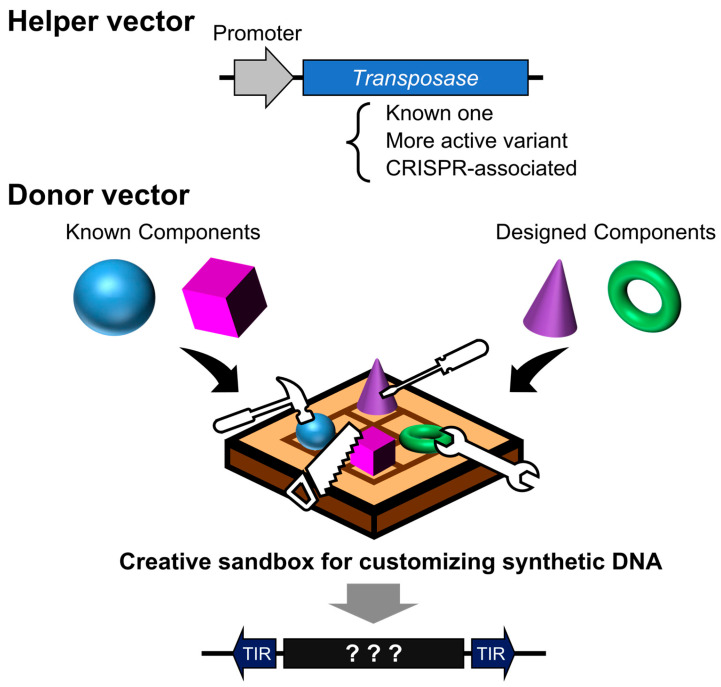
Design contest using transposon vectors as programmable insertion tools. A helper vector facilitates the genomic integration of designed DNA sequences, either directionally or at random. The donor vector serves as a design canvas—its sequence can encode existing systems, novel proteins, or functional elements. The outcome varies dramatically depending on how cleverly the DNA sequence is designed.

Nevertheless, it is likely that the examples presented here have yet to fully realize the latent potential of the transposon system. Ongoing efforts to identify novel transposases and enhance their activity are expected to further improve insertion efficiency and expand the diversity of targeting preferences. The integration of advances in DNA synthesis and protein structural prediction tools has made it possible to design sequences with ever-greater precision. As such, we may be entering an era reminiscent of an invention contest (Figure 5)—one where not only sensitivity and specificity are optimized through integration with other technologies, but where even the most unexpected and unconventional ideas may be translated into transformative innovations through the design of synthetic DNA sequences.

The ever-evolving transposon system is poised to continue contributing significantly to gene function analysis, pathway elucidation, target discovery, drug evaluation, and therapeutic development. Looking ahead, the demonstrated utility of cut-and-paste mechanisms suggests that the discovery of transposons with entirely different properties—such as methylation-guided insertional targeting and the ability to leave designer footprints upon integration—could pave the way for even more groundbreaking technologies.

In this light, transposon research—both in terms of fundamental biology and applied methodology—is expected to remain an area of vigorous activity. Its future impact will likely extend beyond biotechnology and medicine, offering deeper insights into genome structure and the mechanisms of genome evolution.

## Abbreviations

The following abbreviations are used in this manuscript:

*BDKRB2*	Bradykinin receptor B2
BiFC	Bimolecular fluorescence complementation
*BiP*	Binding immunoglobulin protein
*Blm*	Bloom syndrome homologue
cAMP	Cyclic adenosine monophosphate
Cas	CRISPR-associated protein
CHO	Chinese hamster ovary
*c-Myc*	Cellular myelocytomatosis oncogene
*CYP24A1*	Cytochrome P450 family 24 subfamily A member 1
ES	Embryonic stem
GFP	Green fluorescent protein
*LEDLGF*	Lens epithelium-derived growth factor
*LINC-PINT*	Long intragenic non-coding RNA p53-induced transcript
LTR	Long terminal repeat
mKG	Monomeric Kusabira green
NF-κB	Nuclear factor kappa-light-chain-enhancer of activated B cells
*OSBPL9*	Oxysterol binding protein-like 9
TIR	Terminal inverted repeats
*TISPL*	Transcript induced by stressors from LINC-PINT locus
*TSKU*	Tsukushi, small leucine-rich proteoglycan
TSS	Target site sequence
UAS	Upstream activating sequence
UTR	Untranslated region
